# CdSe/ZnS Quantum Dots trigger DNA repair and antioxidant enzyme systems in *Medicago sativa* cells in suspension culture

**DOI:** 10.1186/1472-6750-13-111

**Published:** 2013-12-20

**Authors:** Ana R Santos, Ana S Miguel, Anca Macovei, Christopher Maycock, Alma Balestrazzi, Abel Oliva, Pedro Fevereiro

**Affiliations:** 1 Biomolecular Diagnostics Laboratory, Instituto de Tecnologia Química e Biológica, Universidade Nova de Lisboa, Apartado 127, 2781-901 Oeiras, Portugal; 2 Plant Cell Biotechnology Laboratory, Instituto de Tecnologia Química e Biológica, Universidade Nova de Lisboa, Apartado 127, 2781-901 Oeiras, Portugal; 3 Organic Synthesis Laboratory, Instituto de Tecnologia Química e Biológica, Universidade Nova de Lisboa, Apartado 127, 2781-901 Oeiras, Portugal; 4 Department of Biology and Biotechnology, via Ferrata 1, 27100 Pavia, Italy; 5 Universidade de Lisboa, Faculdade de Ciências, 1749-016 Lisboa, Portugal

**Keywords:** CdSe/ZnS quantum dots, 3-Mercaptopropanoic acid, Plant cells, Medicago sativa, Cytotoxicity, Genotoxicity

## Abstract

**Background:**

Nanoparticles appear to be promising devices for application in the agriculture and food industries, but information regarding the response of plants to contact with nano-devices is scarce. Toxic effects may be imposed depending on the type and concentration of nanoparticle as well as time of exposure. A number of mechanisms may underlie the ability of nanoparticles to cause genotoxicity, besides the activation of ROS scavenging mechanisms. In a previous study, we showed that plant cells accumulate 3-Mercaptopropanoic acid-CdSe/ZnS quantum dots (MPA-CdSe/ZnS QD) in their cytosol and nucleus and increased production of ROS in a dose dependent manner when exposed to QD and that a concentration of 10 nM should be cyto-compatible.

**Results:**

When *Medicago sativa* cells were exposed to 10, 50 and 100 nM MPA-CdSe/ZnS QD a correspondent increase in the activity of Superoxide dismutase, Catalase and Glutathione reductase was registered. Different versions of the COMET assay were used to assess the genotoxicity of MPA-CdSe/ZnS QD. The number of DNA single and double strand breaks increased with increasing concentrations of MPA-CdSe/ZnS QD. At the highest concentrations, tested purine bases were more oxidized than the pyrimidine ones. The transcription of the DNA repair enzymes Formamidopyrimidine DNA glycosylase, Tyrosyl-DNA phosphodiesterase I and DNA Topoisomerase I was up-regulated in the presence of increasing concentrations of MPA-CdSe/ZnS QD.

**Conclusions:**

Concentrations as low as 10 nM MPA-CdSe/ZnS Quantum Dots are cytotoxic and genotoxic to plant cells, although not lethal. This sets a limit for the concentrations to be used when practical applications using nanodevices of this type on plants are being considered. This work describes for the first time the genotoxic effect of Quantum Dots in plant cells and demonstrates that both the DNA repair genes (*Tdp1β*, *Top1β* and *Fpg*) and the ROS scavenging mechanisms are activated when MPA-CdSe/ZnS QD contact *M. sativa* cells.

## Background

Nanoparticles offer many technological solutions since they are valuable as carriers, coaters, repellents, screens and conductors. Nanoparticles may also be useful as nanosensors, cell-imaging devices and smart delivery systems and appear to be promising devices for application in the agriculture and food industries.

While the full potential of new nanomaterials is still far from being explored, their impact on living systems shows that different type of toxic effects may be imposed, depending on the type and concentration of nanoparticle as well as the time of exposure, among other factors. Nanotoxic effects have been detected at relatively high, in many cases unrealistic, particle concentrations and associated with cell death, but subtler effects that arise at lower concentrations without necessarily causing cell death also need to be considered. In particular, a number of mechanisms were envisage underlying the ability of nanoparticles to cause DNA damage [1]. It was found that quantum dots can damage DNA by factors such as surface coatings [2].

Quantum dots (QD) are fluorescent semiconductors extensively used in biological studies [3]. CdSe-core QD are often used for these studies because they are easily prepared, have size tunable properties, a narrow emission band and a broad absorption spectrum. They can be coated with ZnS in order to protect the core from oxidation and other degradation processes that could release Cd ions into the medium [4]. 3-Mercaptopropanoic (MPA) coated CdSe/ZnS quantum dots (MPA-CdSe/ZnS QD) are readily prepared by the ligand exchange/phase transfer method [5]. They are small and stable water soluble QD due to the carboxyl groups [6] and this facilitates their uptake by biological systems. The mercapto group of the MPA provides a suitable ligand for attachment to the Lewis acidic Zn atoms on the QD. However, this is a relatively weak bond and some dissociation could occur [7].

In a previous study, we showed that *Medicago sativa* cells growing in suspension culture accumulated MPA-CdSe/ZnS-QD in the cytosol and particularly in the nucleus 8 [4]. This accumulation induced the production of undifferentiated ROS in a dose dependent manner and it was shown that a maximum concentration of 10 nM should be cyto-compatible [8]. We also showed that cell suspension cultures exposed to 100 nM of MPA-CdSe/ZnS-QD during 48 hours did not show any noticeable production of superoxide radicals (O_2_^–^•), and the production of H_2_O_2_ was far less than 10 nM, if any [8].

Little information has been found in the literature on the expression and activity of plant detoxifying enzymes and DNA repairing enzymes in response to contact with nanoparticles. Plants respond to toxicity by producing ROS that trigger the activation of ROS scavenging mechanisms. These mechanisms include the superoxide dismutase (SOD) enzyme, the water-water cycle, the ascorbate-glutathione cycle (AGC), the glutathione peroxidase cycle and the catalase (CAT) enzyme [9]. Most reports on the effect of Cd on the activity of these enzymes indicate that there is a decrease or no variation in the activity of these enzymes when plants were subjected to concentrations in the micromolar range [10].

Genotoxic effects have been reported when nanoparticles interact with living systems. Silver nanoparticles exhibited cytotoxicity by decreasing the mitotic index in a dose dependent manner in root tips of *Allium cepa*[11]. It was also reported that Cd damaged nucleoli in root tip cells of *A. cepa*[12] and altered the synthesis of RNA and inhibited ribonuclease activity in rice [13]. It was demonstrated that 0.5 nM of non-coated CdSe/ZnS QD cause DNA fragmentation and nicking in cell-free systems [14]. Very high doses of Mercaptoacetic CdSe-QD undoped and doped with cobalt induce genotoxicity in mouse tissues [15], but so far there are no reports on the genotoxicity of MPA-CdSe/ZnS QD on plant cells.

In this work, the cyto- and genotoxic effects of MPA-CdSe/ZnS QD in *Medicago sativa* cells in suspension culture were analyzed. It was shown that some of the ROS scavenging mechanisms are active at the cellular level, preventing the accumulation of some specific ROS when cells were exposed to these QD. Moreover, it was demonstrated that extensive DNA damage occurs when a 100 nM solution of MPA-CdSe/ZnS QD is placed in contact with plant cells and that the expression of the DNA repair genes *Top 1* and *Tdp* is activated by the stress imposed by this type of nanoparticles.

## Results and discussion

### MPA-CdSe/ZnS QD trigger the activity of antioxidant enzymes

**Figure 1:**
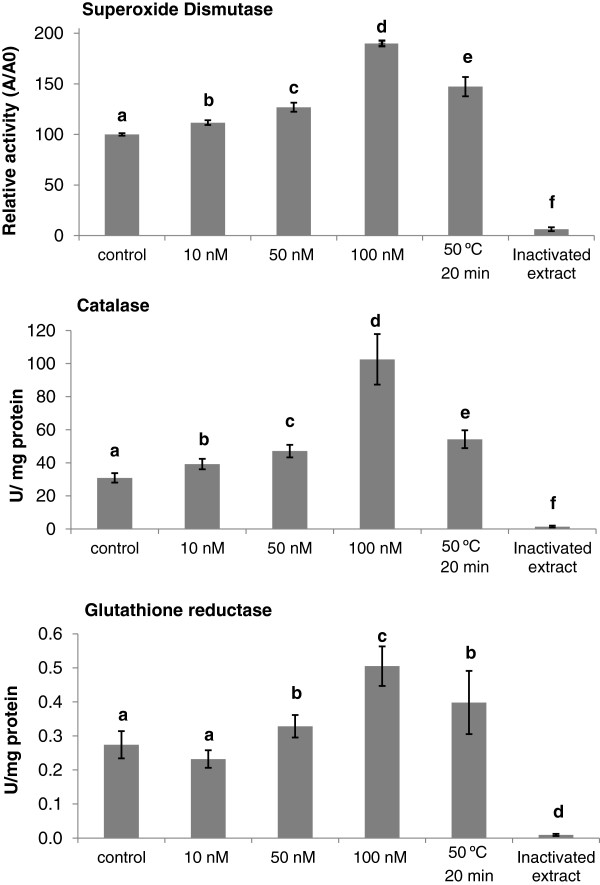
**Effect of MPA-CdSe/ZnS QD on the activities of SOD, CAT and GR.** Enzymatic activity of extracts of cell suspension cultures treated with 0, 10, 50 or 100 nM MPA-CDSE/ZNS QD for 48 hours. SOD is expressed in relative activity, A/A0, where A is the measured enzyme activity for the cells in the presence of MPA-CdSe/ZnS QD and A0 is the enzyme activity of the control. CAT and GR activities are expressed as U mg^-1^ protein. Bars indicate the standard deviation of mean values. Values with different letters are significantly different at p ≤ 0.01.

Knowing that plants exposed to high temperatures increase their anti-oxidant activity [16-18] we have established a positive control for the triggering of antioxidant enzymes. For that, we used a heat shock treatment that involved exposing the cell cultures to 50°C for 20 minutes. Under these conditions, an increase of about 50% in the activity of SOD, CAT and GR was recorded (Figure 1, a, b and c).

SOD activity increased 12%, 27% and 88% when *M. sativa* cells were exposed to 10, 50 and 100 nM of MPA-CdSe/ZnS QD respectively (Figure 1a). The interaction of MPA-CdSe/ZnS QD with plant cells triggers SOD activity, and this may explain why, in a previous study [8], we could not detect O_2_^–•^ accumulation when *M. sativa* cells were exposed to MPA-CdSe/ZnS QD. Within a cell, SODs constitute the first line of defence against ROS, catalyzing the dismutation of O_2_^–•^. Oxygen activation may occur in different compartments of the cell where an electron transport chain is present, such as the cytosol, mitochondria, chloroplasts, peroxisomes and glyoxysomes [19].

CAT activity increased by 8%, 16% and 72% of CAT when *M. sativa* cells were exposed to 10, 50 and 100 nM of MPA-CdSe/ZnS QD respectively (Figure 1b). This significant increase in the activity of CAT suggests a constant detoxification of H_2_O_2_ when *M. sativa* cells are exposed to MPA-CdSe/ZnS QD. Catalase is active only at relatively high H_2_O_2_ concentrations. Low levels of H_2_O_2_ are eliminated by ascorbate peroxidases (APX) and other peroxidases with the aid of various reducing metabolites such as ascorbate and glutathione [20].

Glutathione reductase (GR) activity increased by 5% and 23% when *M. sativa* cells were exposed to 50 and 100 nM solutions of MPA-CdSe/ZnS QD respectively (Figure 1c), while 10 nM MPA-CdSe/ZnS QD induced a GR activity that was not significantly different from the control.

It is possible that the increase in the activity of SOD, CAD and GR could be due to the liberation of Cd ions following MPA-CdSe/ZnS QD degradation. We did not observe this degradation. Kirchner et al. [4] showed that poisoning of normal rat kidney fibroblasts by MPA-CdSe/ZnS particles due to the release of Cd^2**+**^ ions only starts at concentrations of around 6 μM of surface Cd atoms. Neither 1 nor 10 μM Cd ions inhibited the growth of Tobacco BY2 cells in cell suspension cultures and 100 μM Cd induces a decrease of SOD and CAD [21]. In fact, a decrease, and not an increase, of the activity of antioxidant enzymes has been associated with Cd toxicity in different plant species [22-25].

As seen for other oxidative stresses in plants, plant cells respond to the presence of quantum dots by mobilizing ROS scavenging mechanisms to protect the cells from activated oxygen forms. This activation seems to be dose dependent and serves to prevent the accumulation of H_2_O_2_ and O_2_^–•^ when cells are exposed to MPA-CdSe/ZnS QD concentrations between 10 and 100 nM.

### MPA-CdSe/ZnS QD induce DNA damage in exposed plant cells

Four different versions of the Comet assay were used to estimate the range and type of genotoxicity imposed by MPA-CdSe/ZnS QD in *M. sativa* cells: the neutral version, useful to assess DNA double strand breaks (DSBs); the alkaline/neutral version (A/N) that detects mainly DNA single strand breaks (SSBs); the A/N version followed by an enzymatic treatment with formamidopyrimidine DNA glycosylase (FPG), to evaluate the extent of purine base oxidation; and the A/N version followed by the enzymatic treatment with Endonuclease III (EndoIII) to determine the amount of oxidized pyrimidine bases. The two enzymes remove the oxidized bases and generate a DNA strand break at the position of the excised base that can be detected via the comet assay [26].

**Figure 2:**
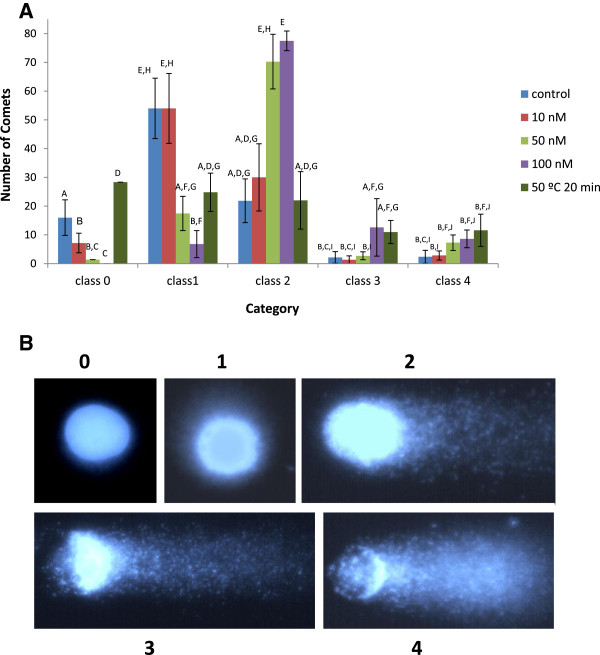
**Histogram of comet distribution. A)** Histogram of comet distribution for cell suspension cultures added with 0, 10, 50 and 100 nM of MPA-CdSe/ZnS QD and subjected to 50°C during 20 min based on the comet score with the A/N version. **B)** Images of comets that represent the 0–4 classes for visual scoring.

Figure 2A shows the A/N Comet assay histogram distribution of cell suspension cultures added to 10, 50 and 100 nM solutions of MPA-CdSe/ZnS QD, or heated at 50°C for 20 minutes. Figure 2B shows images of comets that represent the five classes used for visual scoring. The control shows that 77% of the comets fall into class 1 and 2 while only 4% fall into classes 3 and 4. This may be considered a basal level of damage that may reflect the impact of protoplastization. Except for the 10 nM concentration, an increment of MPA-CdSe/ZnS QD induces an increase in the frequency of comets in the higher classes. When cells are exposed to 100 nM MPA-CdSe/ZnS QD, 78% of the comets fall into class 2 and 13% and 8% of the comets fall in class 3 and 4, respectively. Heat-treated cells present 25% of comet frequencies in classes 0, 1 and 2 and 10% in class 3 and 4. These results show that stressed plant cells undergo DNA SSBs and that increasing concentrations of MPA-CdSe/ZnS QD increment the amount of damage.

**Figure 3:**
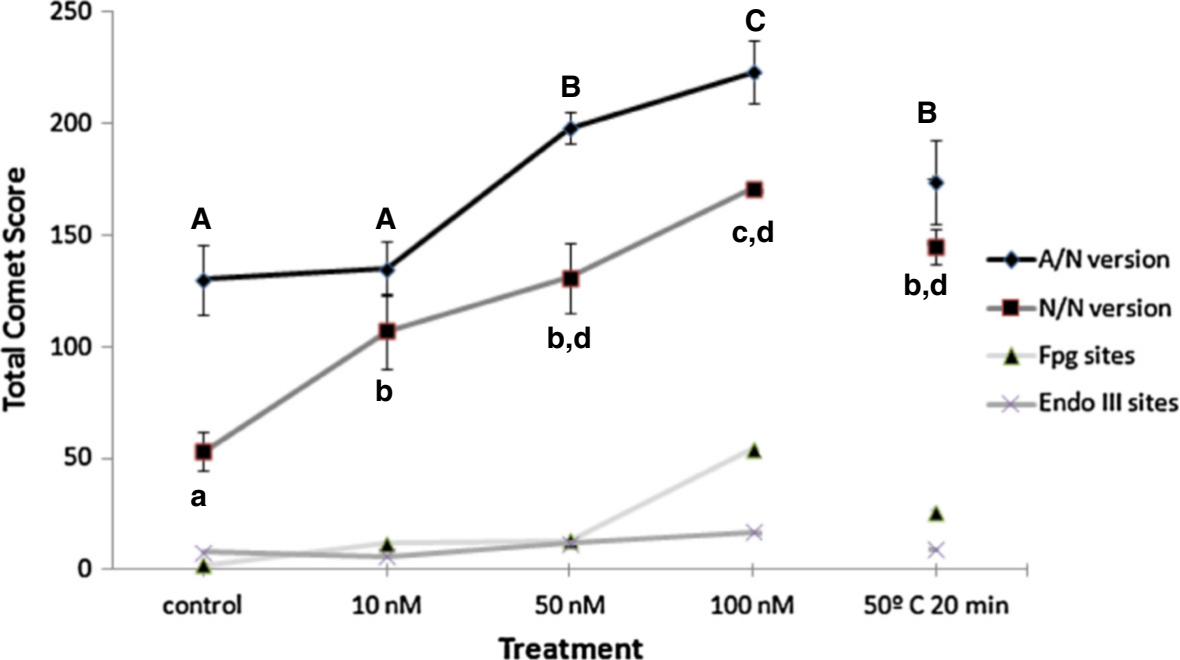
**DNA damage in Medicago sativa cells in suspension cultures.** DNA damage in cell suspension cultures treated with 0, 10, 50 and 100 nM MPA-CdSe/ZnS QD at room temperature for 48 hours or at 50°C for 20 minutes, under alkaline unwinding and neutral electrophoresis (A/N), under neutral incubation and neutral electrophoresis (N/N), and also by incubation with lesion-specific FPG or EndoIII enzymes. Results are expressed as mean values with the standard deviation. One –way ANOVA P < 0.0001. Values with different letters are significantly different at p ≤ 0.01 with Tukey test.

The results of the four variants of the Comet assay were plotted together in Figure 3. Strikingly, contact with 10 nM of MPA-CdSe/ZnS QD induced an increase in the number of DSBs when compared to the control, contrasting with the results obtained for the single strand break analysis. At the highest MPA-QD concentration tested purine bases were more oxidized than the pyrimidine ones.

The accumulation of SSBs and oxidative induced base lesions can lead to DSBs, considered the most lethal type of DNA oxidative damage [26]. Compared with other types of DNA damage, DSBs are intrinsically more difficult to repair and as little as one DSB lesion in the DNA can kill the cell if the lesion deactivates a critical gene. SSBs and oxidatively induced DNA base lesions are known to block DNA transcription and replication processes, resulting in accelerated cytotoxicity and genomic instability [26]. It seems that even 10 nM MPA-CdSe/ZnS QD may induce DNA double strand breaks in plant cells, being potentially deleterious and that the increment of nanoparticles induces an increase in genotoxicity.

### MPA-CdSe/ZnS QD up-regulate DNA repair and antioxidant defence genes

Oxidative DNA damage is typically associated with the accumulation of 7,8-dihydro-8-oxoguanine (8-oxo-dG), an oxidized form of guanine. The 8-oxo-dG is highly mutagenic frequently inducing mispairs with the incoming dAMP during DNA replication and causing G:C to T:A transversions. The Base Excision Repair (BER) is responsible for recognizing and excising damaged bases by a multi-step process using different enzymes, such as DNA glycosylases, AP endonucleases or DNA ligases. Formamidopyrimidine DNA glycosylase (FPG) is a DNA glycosylase/AP lyase enzyme involved in the repair of oxidized purines such as 8-oxo-dG and imidazole-ring opened purines (FapyA, FpyG) [27]. Initially the presence of FPG was considered a unique feature of prokaryotes, but recently it has also been detected in plants [28-30].

**Figure 4:**
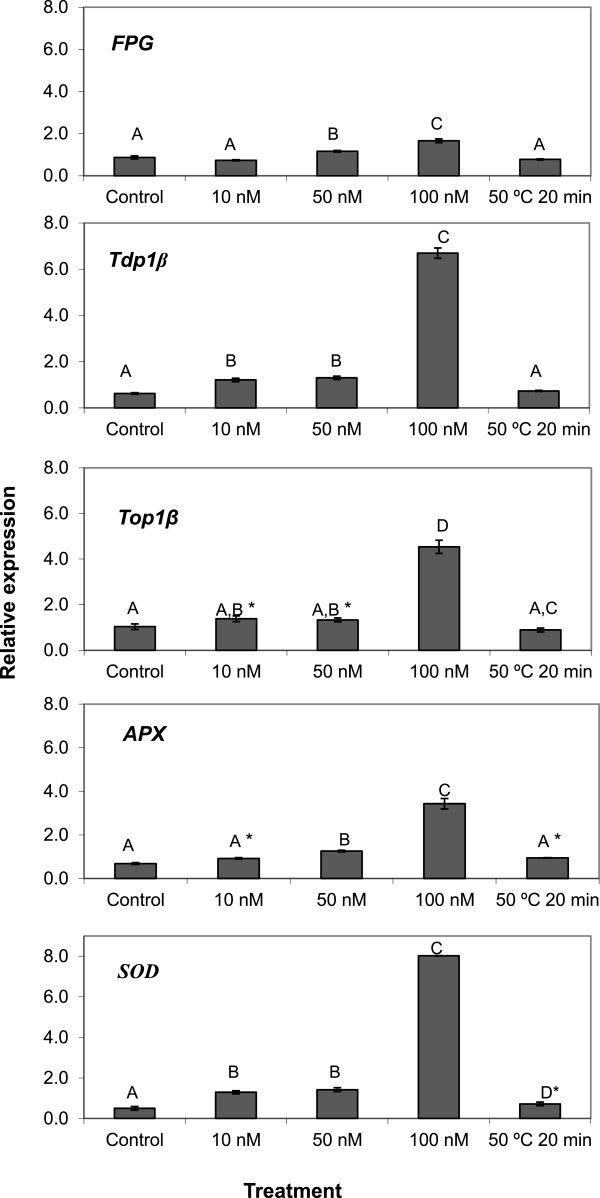
**Expression of *Tdp1 *β, *Top1*β, Fpg, SOD and APX genes in *Medicago sativa *cells treated with MPA-CdSe/ZnS QD.** Expression of *Tdp1* β, *Top1*β, Fpg, SOD and APX genes on cell suspension cultures of *M.sativa* treated for 48 hours with 0, 10, 50 and 100 nM of MPA-CdSe/ZnS QD and at 50°C for 20 minutes. For each treatment, the data represents the mean values of three independent replications. One –way ANOVA P < 0.0001 for *Tdp1b*, Fpg, APX and SOD and P < 0.01 for *Topo1b*. Tukey test P < 0.01 except for *P < 0.05.

The transcript accumulation of the FPG gene was evaluated in *M. sativa* cell suspension cultures exposed to 10, 50 and 100 nM MPA-CdSe/ZnS QD. Changes in the expression levels of the FPG gene were observed during the induced treatments (Figure 4). An up-regulation was observed when the higher QD concentrations (50 and 100 nM) were used (0.7-fold and 2.0-fold, respectively), which is in agreement with the Comet results: cells tend to respond to a genotoxic effect by increasing the expression of FPG to increase the enzyme activity.

Tyrosyl-DNA phosphodiesterase (Tdp1) is a key enzyme that hydrolyzes the phosphodiester bond between the tyrosine residue of DNA topoisomerase I (topo I) and the DNA 3′-phosphate, and thus it is involved in the repair of topoisomerase I – mediated DNA damage [31]. Macovei et al. [32] reported on the presence of a *Tdp1* gene family (*Tdp1α* and *Tdp1β*) in *M. truncatula* and demonstrated its involvement in oxidative stress responses while Lee et al. [33] isolated Tdp1-depleted *Arabidopsis* mutants that exhibited a dwarf phenotype and cell death events, suggesting that this enzyme plays a decisive role during plant development.

The accumulation of transcripts of the β isoforms of *Tdp1* and *Top1* was evaluated in *M. sativa* cell suspension cultures added to 10, 50 and 100 nM MPA-CdSe/ZnS QD. Results (Figure 4) show an increase in the transcript accumulation of both *Tdp1β* and *Top1β* mRNAs. In the case of *Tdp1β,* a 2.0-fold increase was observed at 10 nM and 50 nM, while the addition to 100 nM QD induced a 7.0-fold transcript accumulation. The expression of the *Top1β* gene did not show a significant change when 10 nM and 50 nM of Cd were added while when 100 nM was added a 4.0-fold increase was observed.

This is the first time that the expression of genes of DNA repair enzymes has been evaluated with nanoparticles in contact with plant cells. The over accumulation of transcripts of *FPG*, *Tdp1β* and *Top1β* shows these nanoparticles are exerting a genotoxic effect that the cells try to counteract by increasing the expression of these genes. This is corroborated by the data obtained from the Comet assays, that show that even 10 nM of MPA-CdSe/ZnS QD may induce a genotoxic response by plant cells. The fact that the expression of *APX* and *SOD* genes is also up-regulated by the nanoparticles (Figure 4), mostly at the highest concentrations, is in agreement with the results obtained for the antioxidant enzyme activities during a previous study [8]. The balance between ascorbate peroxidase and superoxide dismutase activity in cells is considered to be crucial for determining the steady-state level of reactive oxygen species [34]. These enzymatic antioxidant defences protect the cells by directly scavenging hydrogen peroxide and superoxide radicals, converting them into less reactive species [35].

## Conclusions

Although not lethal, concentrations as low as 10 nM of MPA-CdSe/ZnS QD may be cytotoxic and genotoxic to plant cells. This sets a limit for the concentrations to be used when carrying out experiments on plants using nanoparticles of this type.

As previously reported [8], when in contact with the plant cell suspensions, some nanoparticle aggregation was observed. At 10 nM this occurrence is small, but is amplified at higher concentrations. Aggregation may mask an even higher level of stress caused by these nanoparticles at higher concentrations than 10 nM, preventing their absorption into cells.

*M. sativa* cells responded to the oxidative stress caused by the addition of MPA-CdSe/ZnS QD by activating their antioxidant enzyme systems. In this study, three antioxidant enzymes: SOD, CAT and GR were activated within 48 hours of MPA-CdSe/ZnS QD exposure, preventing over-accumulation of H_2_O_2_ and O_2_^–•^, as shown previously [8]. Higher concentrations of MPA-CdSe/ZnS QD may induce the accumulation of ROS that are able to damage the plasma membrane, mitochondria and nucleus.

Cells adapt to the imposed stress by up-regulating antioxidant and/or repair systems. This may protect them against damage to some extent, or sometimes even overprotect them; the cells are then resistant to higher levels of oxidative stress imposed subsequently [36].

This is the first report on the genotoxic effects of MPA-CdSe/ZnS QD in plant cells and demonstrates that both the DNA repair genes (*Tdp1β*, *Top1β* and *FPG*) and the ROS scavenging mechanisms are activated when these QD interacts with *M. sativa* cells.

## Methods

### Synthesis and characterization of QD

3-Mercaptopropanoic acid coated CdSe/ZnS QD were synthesized, solubilised and characterised according to Miguel et al. [5]. In brief, MPA-CdSe/ZnS QD were obtained by the phase transfer method and the resultant water-soluble QD were purified and concentrated using a Sartorius Vivaspin 6 tube (cut-off 10KDa) at 7500 g.

For the characterisation of the synthesized CdSe/ZnS core-shell QD, Transmission Electron Microscopy (TEM) was used. Low-resolution images were obtained using a JEOL 200CX traditional TEM operating at an acceleration voltage of 200 kV. Dynamic Light Scattering (DLS) analysis was performed using a Zetasizer Nano ZS dynamic light scatterer from Malvern Instruments. The water-soluble QD had a hydrodynamic diameter of 13.5 nm and zeta potential of −46.5 mV. The concentration of the stock solution was determined as in [5] using the spectrophotometric method of Yu et al. [37,38]. Appropriate dilution of this stock solution afforded the solutions used in this study.

### Cell suspension culture treatments

*M. sativa* cell suspension cultures previously established [8] were used and maintained in an orbital shaker at 110 rpm (Innova 4900, New Brunswick Scientific, Germany) in the dark at 24°C. A MPA-CdSe/ZnS QD stock solution was added to the cell suspension cultures at day 3 of culture (beginning of exponential phase) to obtain the different final concentrations (0, 10 nM, 50 nM and 100 nM). After 48 hours of incubation cells were harvested for RNA or enzyme extraction and frozen at -80°C or used directly for the Comet assays. Cell suspension cultures heat-treated at 50°C for 20 min were used as an abiotic stress control.

### Antioxidant enzyme activity

#### Enzyme extraction

The following steps were carried out at 4°C unless otherwise stated. The *in vitro* cultured *Medicago sativa* cells (about 500 mg of fresh weight) were homogenized in a mortar with 2 mL of 100 mM Tris–HCl buffer (pH 7.5) containing 1 mM ethylenediaminetetraacetic acid (EDTA), 3 mM DL-dithiothreitol, 0.2% Triton X-100 and 2% (w/v) insoluble PVPP. The homogenate was centrifuged at 12000  *g* for 30 min and the supernatant was stored in separate aliquots at -80°C, for CAT, GR, SOD and protein quantification.

For the enzyme assays three types of controls were used: a stress control (heated cells), a control with no treatment and a negative control consisting of a boiled extract of the non treated cells (inactivated enzyme).

#### Protein quantification

Protein concentration was quantified spectrophotometrically at 595 nm according to the Bradford method [39] with BSA as a standard.

Protein quantification and all enzyme activities were measured using an Ultrospec 4000 UV/Visible Spectrophotometer (Pharmacia Biotech).

#### Quantification of Superoxide Dismutase activity

Total SOD activity was quantified according to the modified method described by Rubio et al. [40], measuring the increase in absorbance at 550 nm for 2 minutes (10 seconds interval) in a 1 mL solution containing 0.5 mM xanthine, 0.05 mM ferricytochrome-C, 0.1 mM EDTA, 0.01U of xanthine-oxidase and 0.05 mL of enzyme extract in 100 mM potassium phosphate buffer (pH 7.5). The enzymatic activity was estimated as the quantity of enzyme necessary for the inhibition of 50% of ferricytochrome-C reduction per minute under the assay conditions [41]:

$$\begin{array}{c} \mathrm{Units}/\mathrm{mg}\;\mathrm{protein}=\left(\%\;\mathrm{inhibition}/50\%\right)\\ \;*\left(1/v\right)\mathrm{mg}\;\left(\mathrm{of}\;\mathrm{total}\;\mathrm{protein}\right) \end{array}$$

Where: % inhibition = (ΔAbs control‒ΔAbs sample)/ΔAbs control*100; 50% = inhibition of the rate of cytochrome C reduction; v (volume of enzyme extract) = 0:05 mL

#### Quantification of Catalase activity

Total CAT activity was measured as described in [42]. Briefly, the decrease in absorbance was measured at 240 nm for 2 minutes (10 seconds intervals), in a 1 mL solution containing 10 mM H_2_O_2_ in 50 mM phosphate buffer (pH 7.5). CAT enzymatic activity was defined as the consumption of 1 μmol H_2_O_2_ per minute per ml at room temperature, under the assay conditions, according to the following equation:

$$\left(\mathrm{\Delta Abs}/\mathrm{\Delta T}\right)*\left(1/\epsilon \right)*\left(1/L\right)*\left(1/v\right)/\mathrm{mg}\;\mathrm{of}\;\mathrm{total}\;\mathrm{protein}$$

Where ϵ H_2_O_2_ = 0.00394 μmol ^−1^ mm^−1^; L = 10 mm; v = 0.037 mL.

#### Quantification of Glutathione reductase activity

GR activity was quantified based on the increase in absorbance at 412 nm (10 seconds interval during 2 minutes) when 5.5′-dithiobis (2-nitrobenzoic acid) (DTNB) was reduced by GSH [43]. The 1 mL reaction mixture contained 100 mM potassium phosphate buffer at pH 7.5, 1 mM EDTA, 0.75 mM DTNB, 0.1 mM NADPH and 1 mM GSSG. The components of the reaction mixture were added in the stated order and the reaction was initiated by the addition of GSSG. The activity of the enzyme was expressed in U/mL*mg protein wherein unit activity is the amount of enzyme which reduces 1 mM of GSSG per minute at 24°C under assay conditions:

$$\left(\mathrm{\Delta Abs}/\mathrm{\Delta T}\right)*\left(1/\epsilon \right)*\left(1/L\right)*\left(1/v\right)/\mathrm{mg}\;\mathrm{of}\;\mathrm{total}\;\mathrm{protein}$$

Where ϵ GSSG = 0.62 mL μmol ^−1^ mm^−1^; L = 10 mm; v = 0.05 mL.

### Comet assay

#### Protoplast preparation

Cells from the suspension culture were pelleted by centrifugation at 1000 rpm for 10 min and incubated with a protoplastization solution consisting of 10 mM MES buffer pH 5.8, 10 mM CaCl_2_, 0.4 M mannitol, 1% Macerozyme and 1% Cellulase (for about 1 g of cells 5 mL enzymatic solution was added) at room temperature in the dark for 3–4 hours under gentle agitation. After incubation the protoplasts were sieved through a 90 μm mesh without applying pressure.

200 μL of protoplasts were mixed with 200 μL of 0.75% LMP agarose (at 3°C) and 80 μL aliquots were placed on a microscope slide previously coated with 0.75% agarose. A 22×22 mm glass cover slip was placed on each gel and the slides were allowed to set on ice for a few minutes, the coverslips were then removed. The slides were marked as “control” (protoplasts from cultures with no treatment), “heat treated” (protoplasts treated for 20 min at 50°C), “10 nM, 50 nM or 100 nM” (protoplast from cultures treated with one of the three QD concentrations), “buffer” (protoplasts from cultures treated with one of the three QD concentrations plus enzyme buffer), “FPG” (protoplasts from cultures treated with one of the three QD concentrations plus FPG enzyme) and “Endo III” (protoplasts from cultures treated with one of the three QD concentrations plus Endo III enzyme).

#### Alkaline unwinding/neutral electrophoresis

The modification of the comet assay described by Angelis et al. [44] employs various combinations of neutral and alkaline solutions immediately prior to and during electrophoresis. Exposure of DNA to highly basic conditions prior to electrophoresis under neutral conditions (N/A protocol) allows for the preferential detection of DNA SSBs.

Briefly, cells embedded in agarose were lysed in a Coplin jar for 1 hour in 2.5 M NaCl, 0.1 M EDTA, 10 mM Tris–HCl pH 10, 1% Triton X-100 at 4°C. The slides marked with “buffer”, “FPG” and “EndoIII” were then washed 3 times for 5 minutes at 4°C with enzyme buffer containing 40 mM HEPES, 0.1 M KCl, 0.5 mM EDTA, 0.2 mg/mL BSA, pH 8 adjusted with KOH. After the last wash the excess of liquid was drained with the tissue and the slides were placed on ice. Then 50 μL of enzyme buffer, FPG (10^4^ dilution) or Endo III (10^4^ dilution) were added to the respective gels and covered with a coverslip. The slides were then transferred to a moistening box and incubated at 37°C for 30 min.

During this time the slides marked as “control”, “heat treated” and “10 nM, 50 nM or 100 nM” were kept in the lysis solution. At the end of the incubation period, the coverslips were removed and all the slides were placed in 0.3 M NaOH and 1 mM EDTA, pH approximately 13,0 at 4°C for 20 minutes.

The samples were then neutralized by dipping in a 0.4 M Tris–HCl, pH 7.5 solution, 3 times for 5 minutes at 4°C. The slides were transferred to the electrophoresis tank and placed in TBE (pH 8) for a few minutes and then electrophoresed for 10 min at 25 V, 10 mA at 4°C. After being electrophoresed they were fixed in ethanol 70% 2x5 min and left to dry overnight. 20 μL of 1 μg/mL DAPI was placed on each gel and covered with a coverslip, and scored after 5 min.

#### Neutral incubation/ neutral electrophoresis

DNA unwinding and electrophoresis at neutral pH (pH 7–8) facilitates the detection of double-strand breaks and crosslinks. Under these conditions the total DNA damage is much less pronounced than under alkaline conditions [45].

In brief, slides marked as “control”, “heat treated” and “10 nM, 50 nM or 100 nM” were lysed in the Coplin jar for 1 hour at 4°C in 2.5 M NaCl, 0.1 M EDTA, 10 mM Tris–HCl pH 7.5. They were then equilibrated in TBE 2 times for 5 min and electrophoresed in TBE 10 min at 25 V, 10 mA. They were fixed, stained as above and scored.

#### Scoring for DNA damage

Visual image analyses of DNA damage were carried out in accordance with the described protocol [46]. Slides were examined at 200 X magnification on a Nikon Eclipse TE2000-S (Japan) inverted microscope equipped with a HMX-4 100 W Mercury lamp and UV excitation filter. One hundred randomly selected non-overlapping nucleoids were analyzed by visual inspection giving each comet a value of 0–4 according to the degree of damage. Examples of images of nuclei falling into the different classes are seen in Figure 2B. Two or three slides were evaluated per treatment and each treatment was repeated at least twice.

Images were acquired with an Evolution MP 5.1 megapixel digital CCD Color Camera (Media Cybernetics) controlled by Image Pro Plus 5.0 software (Media Cybernetics).

For the lesion-specific enzymes the standard procedure was used, including a control slide (incubated with buffer alone) in parallel with the slide treated with the enzyme, and to subtract the mean Comet score of the control from the mean score of the slide treated with the enzyme. Net enzyme-sensitive sites constituted the measure of the oxidized bases concerned.

### Real time quantitative Polymerase Chain reaction

#### RNA extraction

The RNA extraction protocol was based on the protocol developed by Chang et al. [47] with some modifications. Frozen cells were ground in the presence of liquid nitrogen in a mortar and the powder was transferred to a 2 mL microcentrifuge tube. The extraction buffer containing 2% CTAB, 2% PVP, 100 mM Tris–HCl pH 8, 25 mM EDTA, 2 M NaCl, 0.5 g/L spermidine and 2% β-mercaptoethanol (added just before use) was heated at 65°C for 10 min in a water bath. 900 μL of this extraction buffer was added to each sample and quickly mixed and vortexed vigorously. Samples were incubated for 15 min at 65°C, then placed on ice for 5 min and 900 μL of chloroform:isoamyl alcohol (CIA) (24:1) was added. Each sample was vigorously vortexed until a unique liquid phase was observed and again placed on ice for 5 min. Samples were then centrifuged for 15 min at 20000 *g*. The CIA extraction was repeated 3 times. The final combined supernatant was placed into a new microcentrifuge tube and 65 μL of 4 M NaOAc (pH 5.2) and 1500 μL of ethanol were added. Each sample was mixed by inversion and allowed to precipitate at -20°C for one hour. The samples were then centrifuged for 30 min at 20000 *g* and the supernatant was carefully decanted. 250 μL of 70% ethanol at 4°C was then added, the mixture centrifuged and the supernatant again discarded. An additional washing with absolute ethanol at 4°C was carried out. The pellet was dried and re-suspended in 50 μL of Milli-Q water and 50 μL of 12 M LiCl added and left to precipitate overnight at -20°C. It was then centrifuged 1 hour at 20000 *g* and the supernatant discarded. The residue was subsequently washed with 70% ethanol and, finally, stored in absolute ethanol. Total RNA was quantified in the Nanodrop1000 spectrophotometer (Thermo Fisher Scientific) and quality assessed by agarose gel electrophoresis. Only the samples with purity (A260/280 ratio) between 1.8-2.0 were used for qPCR.

#### cDNA synthesis and real time quantitative Polymerase Chain Reaction (qPCR)

The total RNA was reversely transcribed into cDNAs using the iScript cDNA Synthesis Kit (Bio-Rad), as indicated by the supplier.

The high degree of sequence identity and remarkably conserved genome structure and function between *Medicago truncatula* (barrel medic) and *M. sativa* (alfalfa) provides the opportunity to use the model legume *M. truncatula* as a surrogate [48-50] to design the oligonucleotide sequences of *Tdp1β*, *Top1β*, *FPG, SOD* and *APX* genes. Primers were designed using the Real-Time PCR Primer Design, GenScript software, (https://www.genscript.com/ssl-bin/app/primer) covering their highly conserved motifs. The primer sequences used are listed in Table 1. The *ELF1α* gene was used as a reference for the qPCR reactions [51].

**Table 1:** Primer sequences

**Gene**	**Forward primer (5′-3′)**	**Reverse primer (5′-3′)**	Efficiency*
*Tdp1β*	GGTTGGTTTGAGCCATCTTT	GCAGGCACATTGTGATTTCT	1.79
*Top1β*	ATACACGTGGGCTATTGTCG	TCACTTGGATGAATGCGTT	1.77
*FPG*	TCCTTTCAATTCGGTATGGC	GCTCCAAACCATCGTCTAGC	1.76
*APX*	AGCTCAGAGGTTTCATCGCT	CGAAAGGACCACCAGTCTTT	1.80
*SOD*	CCTGAGGATGAGACTCGACA	GAACAACAACAGCCCTTCCT	1.72
*ELF1α*	GACAAGCGTGTGATCGAGAGATT	TTTCACGCTCAGCCTTAAGCT	1.90

^*^Efficiency of the primer pair in qPCR.

qRT-PCR was carried out in a Rotor-Gene 6000 PCR apparatus (Corbett Robotics, Australia) by adding 10 μl of SsoFast EvaGreen Supermix (Bio-Rad), 200 ng of cDNA, 0.5pmol of each primer and water to a final volume of 20 μL. After one initial incubation step at 95°C for 30 sec, amplification was performed for 40 cycles with the following profile: denaturation at 95°C for 5 sec, annealing at 60°C for 10 sec and extension at 72°C for 10 sec. Fluorescence data were collected during the extension (72°C) step and the specificity of PCR products was confirmed by performing a melting temperature analysis at temperatures ranging from 55°C to 95°C at intervals of 0.5°C. The PCR products were subsequently run on a 2.5% agarose gel to confirm the presence of a unique band with the expected size. The resulting PCR efficiency and Ct (Treshold Cycle) were used for transcript quantification. The Pfaff method [52] was used for the relative quantification of the transcript accumulation. For all the tested genes and treatments, three independent replicates were performed.

### Statistical analysis

All results are presented as the mean ± standard deviation (SD). The One Way ANOVA test of significance was used to compare the different conditions by Tukey Test (VasserStat Website for Statistical Computation, http://vassarstats.net).

## Abbreviations

CAT: Catalase; DSBs: Double strand breaks; Endo III: Endonuclease III; FPG: Formamidopyrimidine DNA glycosylase; GR: Glutathione reductase; M&S: Murashige & Skoog; MPA: 3-Mercaptopropanoic acid; QD: Quantum Dots; ROS: Reactive oxygen species; SOD: Superoxide dismutase; SSBs: Single strand breaks; Tdp: Tyrosyl-DNA phosphodiesterase; Top: Topoisomerase.

## Competing interests

The authors declare that they have no competing interests.

## Authors’ contributions

ARS, ASM and AM participated equally in the execution of the experiments and wrote the first draft of the manuscript. CM supervised the QD synthesis and contributed to the revision of the manuscript. AB, AO and PF participated in the design and coordination of the study, contributed to the interpretation of data and revision of the manuscript. All authors read, participated in the writing of and approved the final manuscript.
